# miR-155 and miR-21 as Diagnostic and Therapeutic Biomarkers for Ulcerative Colitis: There Is Still a Long Way to Go

**DOI:** 10.3390/biomedicines12061315

**Published:** 2024-06-13

**Authors:** Danusia Onisor, Olga Brusnic, Claudia Banescu, Claudia Carstea, Maria Sasaran, Mircea Stoian, Calin Avram, Adrian Boicean, Alina Boeriu, Daniela Dobru

**Affiliations:** 1 Department of Internal Medicine VII, George Emil Palade University of Medicine, Pharmacy, Science and Technology of Targu Mures, Gheorghe Marinescu Street No. 38, 540136 Targu Mures, Romania; danusia.onisor@umfst.ro (D.O.); alina.boeriu@umfst.ro (A.B.); daniela.dobru@umfst.ro (D.D.); 2 Gastroenterology Department, Mureș County Clinical Hospital, 540072 Targu Mures, Romania; 3 Genetics Department, Center for Advanced Medical and Pharmaceutical Research, George Emil Palade University of Medicine, Pharmacy, Science and Technology of Targu Mures, Gheorghe Marinescu Street No. 38, 540136 Targu Mures, Romania; claudia.banescu@umfst.ro (C.B.); claudia.carstea@umfst.ro (C.C.); 4 Department of Pediatrics III, George Emil Palade University of Medicine, Pharmacy, Science and Technology of Targu Mures, Gheorghe Marinescu Street No. 38, 540136 Targu Mures, Romania; maria-oana.marginean@umfst.ro; 5 Department of Anesthesiology and Intensive Care, George Emil Palade University of Medicine, Pharmacy, Sciences and Technology of Targu Mures, 540139 Targu Mures, Romania; mircea.stoian@umfst.ro; 6 Department of Medical Informatics and Biostatistics, George Emil Palade University of Medicine, Pharmacy, Science and Technology of Targu Mures, Gheorghe Marinescu Street No. 38, 540136 Targu Mures, Romania; 7 Faculty of Medicine, Lucian Blaga University of Sibiu, 550169 Sibiu, Romania; adrian.boicean@ulbsibiu.ro

**Keywords:** microRNAs, ulcerative colitis, irritable bowel syndrome, Clostridioides difficile infection

## Abstract

(1) Elucidating the role of miRNAs (miRs) in ulcerative colitis may provide new insights into disease pathogenesis, diagnosis, treatment, and monitoring We aimed to investigate whether plasma levels of miR-21-5p and miR-155-5p may be used to differentiate between patients with organic disease such as ulcerative colitis (UC) and Clostridioides difficile infection (CDI), and patients with functional disease such as irritable bowel syndrome with diarrhea (IBS-D). (2) Serological samples were collected to quantify miR-155 and -21 expression, which was carried out through quantitative real-time polymerase chain reaction (qRT-PCR), from 84 patients: 34 with acute UC (group 1), 17 with CDI (group 2), and 33 with IBS-D (control group). (3) In this study, we found that the expression levels of miR-155-5p were almost the same for the two conditions and the control group (UC: 4.22 ± 1.61, CDI: 3.94 ± 1.62, IBS-D: 4.26 ± 1.26), with no significant differences either for ΔCt- or for ΔΔCt-derived parameters (*p* = 0.74 and *p* = 0.73, respectively). For miR-21, ΔCt levels presented significantly higher values among the ulcerative colitis group (*p* < 0.01), but the most important expression fold change was noticed in patients with CDI (UC:4.11 ± 8,46, CDI: 4.94 ± 9.68, IBS-D: 2.83 ± 5.41). (4) Circulating miR-155 and miR-21 were upregulated in UC, CDI, and IBS-D, but differentiation was not possible among them. But their involvement in the pathogenesis of the three diseases makes them suitable for improving the accuracy of diagnosis and facilitating the development of personalized treatment strategies.

## 1. Introduction

Ulcerative colitis (UC) is a potentially severe chronic inflammatory condition of the colon, characterized by periods of activity and remission, with incompletely elucidated etiopathogenesis, in which the immune system interacts with microbiota disorders and/or environmental factors in genetically predisposed individuals [1]. The clinical picture in UC is determined by the extent of the disease and the severity of the inflammation, characterized by the presence of diarrhea and rectal bleeding [2]. Clostridioides colitis (CDI) is a severe inflammation of the inner lining of the large intestine, manifesting as an antibiotic-associated colonic inflammatory complication [3], caused by toxins produced by Clostridioides difficile. Research suggests that individuals with UC are about five times more likely to develop CDI compared to those without UC [4]. Additionally, UC patients exhibit a higher prevalence of asymptomatic carriage of Clostridium difficile than the general population [5]. The symptoms of CDI can be similar to those of UC: diarrhea, fever, and abdominal pain [6,7]. On the other hand, irritable bowel syndrome with a predominance of diarrhea (IBS-D) is a functional intestinal disorder characterized by recurrent abdominal pain associated with defecation or changes in intestinal transit [8]. Similar symptoms may appear at the onset of UC or during its evolution [9]. IBS-D is defined according to the Rome IV criteria as being characterized by more than 25% of stools of low consistency (type 6 and 7 on the Bristol scale) and less than 25% of high consistency (type 1 and 2 on the Bristol scale) [10,11]. IBS and IBD show many similarities and have some overlapping pathophysiological mechanisms, such as increased intestinal permeability, altered immune system activation, inflammation, and changes in the intestinal microbiota [8]. UC can begin acutely or insidiously, and making the differential diagnosis of the inaugural episode, as well as subsequent exacerbations, is sometimes very difficult.

To assist clinicians, several markers were outlined: C-reactive protein (CRP) [2,12], fecal calprotectin (FC) [2,13,14], and fecal lactoferrin [2]. While these markers can aid in differentiating between organic and functional conditions [2], they cannot distinguish between UC and infectious colitis (CDI) [2,13]. Therefore, the necessity for new markers has spurred research expansion and the consideration of new molecules, such as microRNAs, which are stable in peripheral blood and play an important, though not yet fully established, role in the pathophysiology of UC [15,16]. MicroRNAs (miRs) are molecules consisting of 18–25-nucleotide non-coding RNA which regulate the expression of post-transcriptional genes [17], with significant implications in cell survival, apoptosis, malignant transformation, and dissemination [16,18]. In the pathogenesis of UC, a significant number of miRs show increased expression in peripheral blood (miRs 16, -21, -155 [18], and miRs-92 [19]) or in tissue (miRs-21, -29a [20], miRs-126, and -31 [21]). For IBS-D, studies have examined miR-155 [22] or miRs-16 and -125-5p [23]. Unfortunately, the target genes of these miRs involved in the UC inflammation cascade are not yet fully elucidated [24]. It is known that miR-155 appears to play a role in the intestinal inflammation of patients with active UC by using different pathways to downregulate the expression of FOXO3a [18,25], or it is upregulated in UC patients, modulating the inflammatory phenotype of IMFs by targeting the suppressor of cytokine signaling 1 (SOCS1) [26]. MiR-21-5p intervenes in intestinal epithelial barrier function, causing the loss of tight junctions in intestinal epithelial cells [15,27].

In this context, firstly, this study aimed to investigate whether plasma levels of miR-21-5p and miR-155-5p can be considered biomarkers for UC and may be used to differentiate between patients with organic disease (UC and CDI) and functional disease (IBS-D). Secondly, it aimed to asses the expression of miR-155-5p and miR-21-5p in UC regarding disease extent and activity.

## 2. Materials and Methods

### 2.1. Study Population and Data Collection

We conducted a prospective case-control pilot study, which included 84 patients (34 with active UC, 17 with CDI, and 33 with IBS-D), selected from the Gastroenterology Clinic at the County Clinical Hospital, Târgu Mureș, Romania. The division of the study groups was based on the presence or absence of lesions in the colonic mucosa. Thus, group 3 or control group consisted of patients without any pathological modifications of the colonic mucosa (IBS-D patients), whereas the study group comprised patients with inflammatory changes of the colonic mucosa (UC patients: group 1; CDI patients: group 2). Inclusion criteria consisted of adult subjects aged 18 years or older with a previous diagnosis of active UC, patients with acute CDI, and patients with IBS-D. The exclusion criteria comprised patients who did not sign the informed consent or declined participation in this study, patients with Crohn’s disease or undifferentiated colitis, those with celiac disease, patients with chronic rheumatological conditions, unbalanced diabetes, chronic obstructive pulmonary disease, ischemic heart disease, chronic renal failure, and cerebrovascular accident, and individuals who had colorectal neoplasia or other malignancies in the previous five years.

The diagnosis of UC was made according to the criteria of the European Crohn’s and Colitis Organizations [2] guidelines and Montreal classification for extension [28]. The severity of UC was assessed using the Ulcerative Colitis Disease Activity Index (UCDAI): mild < 3, moderate between 3 and 7, and severe ˃ 7. The diagnosis of IBS-D was made in accordance with the Rome IV criteria and assessed by using the Bristol scale [10]. Patients with UC and IBS-D underwent a stool ovum and parasite exam, and a fecal culture test including mycology test, and were also tested for Clostridioides Difficile toxins with negative results. CDI and pseudomembranous colitis were diagnosed according to the National Diagnostic Guideline and treatment of Clostridium Difficile infection [7]. Patients with CDI were evaluated endoscopically, after the infection was cured, to exclude other pathological processes. The endoscopic investigations were performed by experienced endoscopists, following current protocols, with short-term sedation using Propofol, under the careful monitoring of an anesthetist. The following data were collected: demographic data, laboratory analyses including blood count, albumin, iron, fecal calprotectin, PCR, and ulcerative colitis disease activity index (UDCAI) score.

### 2.2. Ethics Statement

The study was approved by the Ethics Committees of the County Clinical Hospital, Târgu Mureș (No. 4872/24 May 2022), and the George Emil Palade University of Medicine, Pharmacy, Science and Technology, Târgu Mureș (No. 1804/22 June 2022). All subjects provided informed consent before enrollment in the study.

### 2.3. Serum Sample Collection

Under aseptic precautions, 6 milliliters of venous blood were collected in an EDTA tube from each participant and carefully centrifuged at 4000 rpm for 20 min. The separated plasma was stored at −80 °C until the time of analysis.

### 2.4. RNA Extraction and Reverse Transcription

The total RNA was isolated from the blood samples by using MicroRNA: mirVana miRNA Isolation Kit with phenol (Thermo Fischer Scientific, Waltham, MA, USA). The obtained nucleic acids were stored at −80 °C, until further analysis. Reverse transcription was performed using the TaqMan Advanced miRNA cDNA Synthesis Kit to obtain cDNA for the detection and quantification of miRNA 21-5p and miRNA 155-5p. miRNA expression analysis was performed using the Applied Biosystems (Foster City, CA, USA) 7500 Fast Dx-Real Time PCR system, TaqMan Fast Advanced Master Mix and specific TaqMan hsa-miR-21-5p and TaqMan hsa-miR-155-5p assays (Thermo Fischer Scientific, USA). The analysis was performed in triplicate for each sample. TaqMan hsa-miR-93-5p was utilized as a reference considering its reported stable expression in plasma and serum, as indicated by Song et al. [29], which suggested miR-93 as a suitable reference gene for serum miRNA analysis, especially in cases of gastric cancer.

### 2.5. miRNA Expression Analysis

For both miRNAs, the expression level was calculated using miR-93-5p as the endogenous control. The delta (Δ) cycle threshold (Ct) method was employed, where Ct is defined as the minimal number of cycles needed to produce a fluorescent signal in qRT-PCR reactions. Both ΔCt and ΔΔCt values were calculated in accordance with a previously published method [30]. Subsequently, the 2^−ΔCt^/2^−ΔΔCt^ method was applied only in the case of negative values to avoid the aggregation of sub-unitary values.

### 2.6. Statistical Analysis

Descriptive statistics involved the calculation of mean, frequency, and standard deviation parameters. The Chi square test was used to analyze contingency tables. The Kolmogorov–Smirnov test was employed to assess the distribution pattern of the analyzed data. The mean comparison of unpaired quantitative data was conducted based on the distribution of the analyzed data. For non-Gaussian distributed data, the Mann–Whitney test was used, whereas Student’s *t*-test was applied for variables complying with a Gaussian distribution. The Brown–Forsythe and Welch analysis of variance (ANOVA) tests or the Kruskal–Wallis tests were used for the simultaneous comparison of mean and median of the three data sets. Correlation between miRNA expression and parameters suggestive of ulcerative colitis severity implied the application of the non-parametric Spearman correlation test. To establish the discriminatory power of miRNA expression between the three groups, receiver operating characteristic (ROC) analysis was performed, including the calculation of area under the curve (AUC) values, sensitivity, specificity and cut-off values. A confidence interval of 95% was chosen, indicating that only values under 0.05 were considered statistically significant. The GraphPad Prism vers. 10 software was used for the entire statistical analysis.

## 3. Results

Group 1 consisted of 34 patients with histologically confirmed ulcerative colitis, within 4 years from disease onset, group 2 comprised 17 patients with mild severity CDI, whereas the control group encompassed 33 patients with irritable bowel syndrome, classified according to the latest Rome criteria. We did not register any deaths during the study period. The demographic characteristics of the three groups enrolled in the study are depicted in Table 1. Group 1 was distinguished by a significantly lower mean age compared to the other groups diagnosed with ulcerative colitis (45.91 ± 15.23 SD versus 65 ± 12.51 SD and 59.18 ± 13.86, *p* < 0.01). Therefore, a significantly higher percentage of the active, working population among the ulcerative colitis study group was expected (*p* < 0.01). A higher prevalence of females was observed in patients with irritable bowel syndrome, whereas in group 1, the male-to-female ratio was significantly higher (*p* < 0.01).

The paraclinical assessment of complete blood count parameters focused on hemoglobin levels and leukocyte counts. The lowest hemoglobin levels were found in the ulcerative colitis group (11.32 ± 1.87 SD versus 11.82 ± 1.25 SD and 12.52 ± 0.92 SD, *p* < 0.01), while the highest leukocyte mean counts was observed in group 2 (12,182 ± 2776 SD versus 10,205 ± 2275 SD and 8473 ± 1847 SD, *p* < 0.01). No notable differences in serum iron levels were found between the three groups, but serum albumin levels presented the lowest values among patients with ulcerative colitis and highest levels in those with irritable bowel syndrome (34.47 ± 2.60 versus 36.24 ± 3.54 and 37.21 ± 1.83, *p* < 0.01). Fecal calprotectin levels were significantly higher in group 1 (551.5 ± 424.1 SD, *p* < 0.01), whereas C-reactive protein levels showed the most significant, ascending variation in group 2 (5.18 ± 1.82 SD, *p* < 0.01) (Table 2).

**Figure 1:**
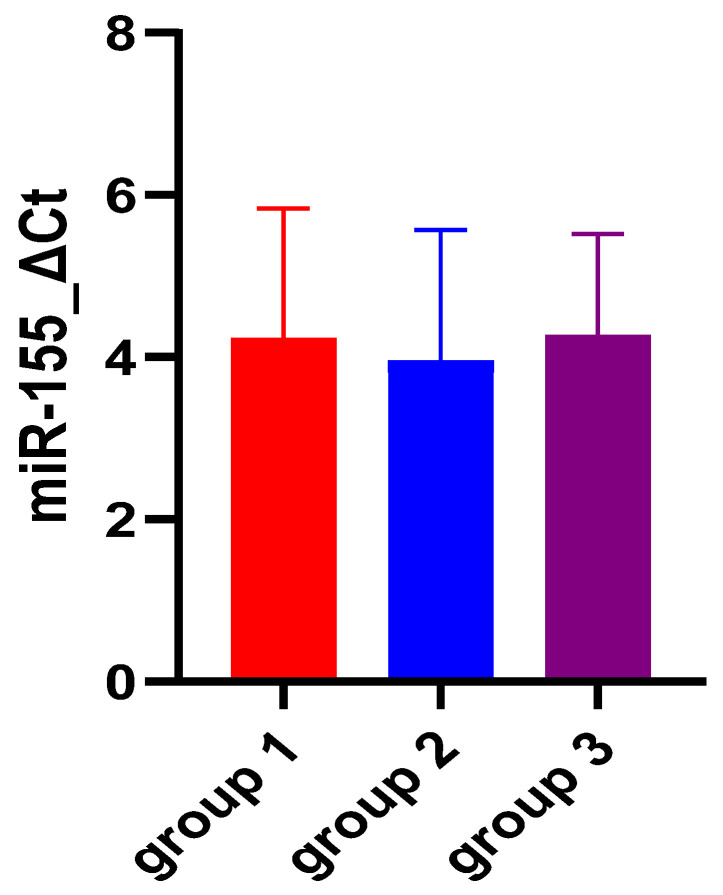
miR-155 expression comparison among the three study groups. Legend: Ct—cycle threshold; group 1—UC patients; group 2—CDI patients; group 3 (control group)—IBS-D patients.

**Figure 2:**
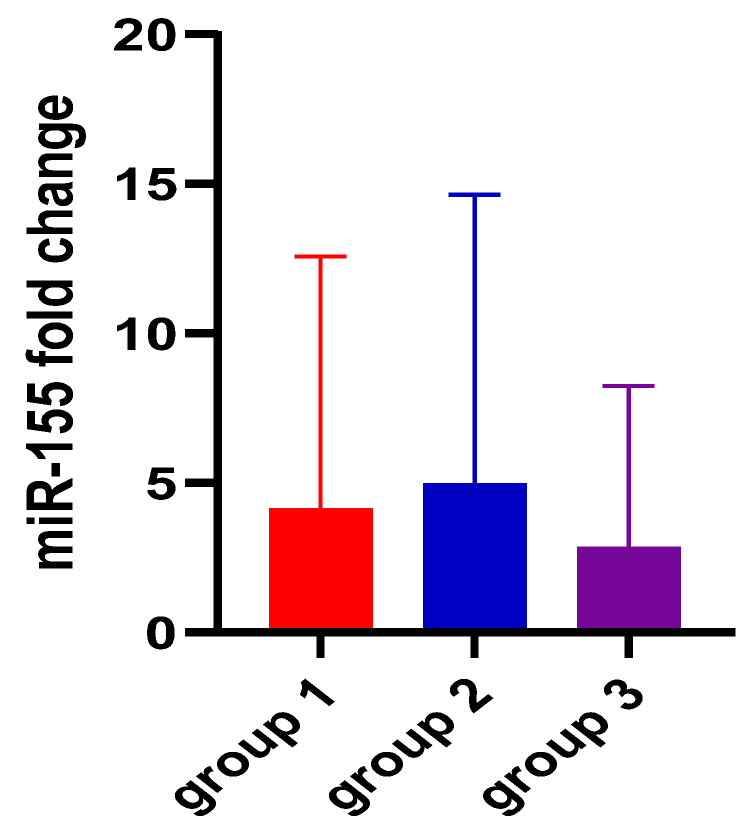
miR-155 2^−ΔΔCt^ fold change among the three groups. Legend: miR-miRNA; group 1—UC patients; group 2—CDI patients; group 3 (control group)—IBS-D patients.

**Figure 3:**
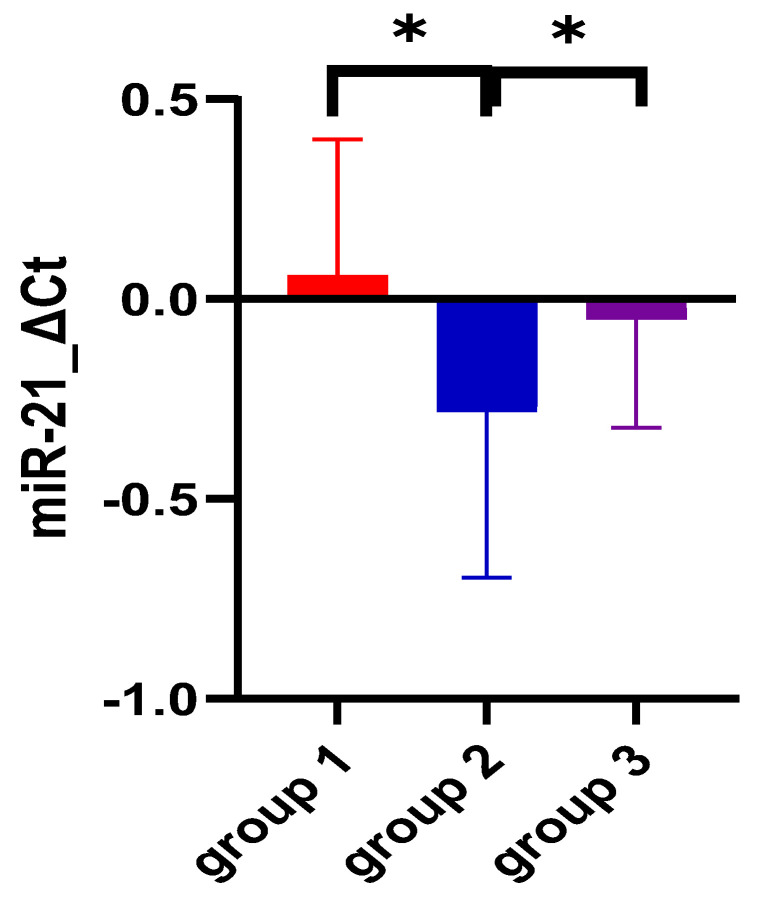
miR-21 expression comparison among the three study groups. Legend: * *p* < 0.05; Ct—cycle threshold; miR—miRNA; group 1—UC patients; group 2—CDI patients; group 3 (control group)—IBS-D patients.

**Figure 4:**
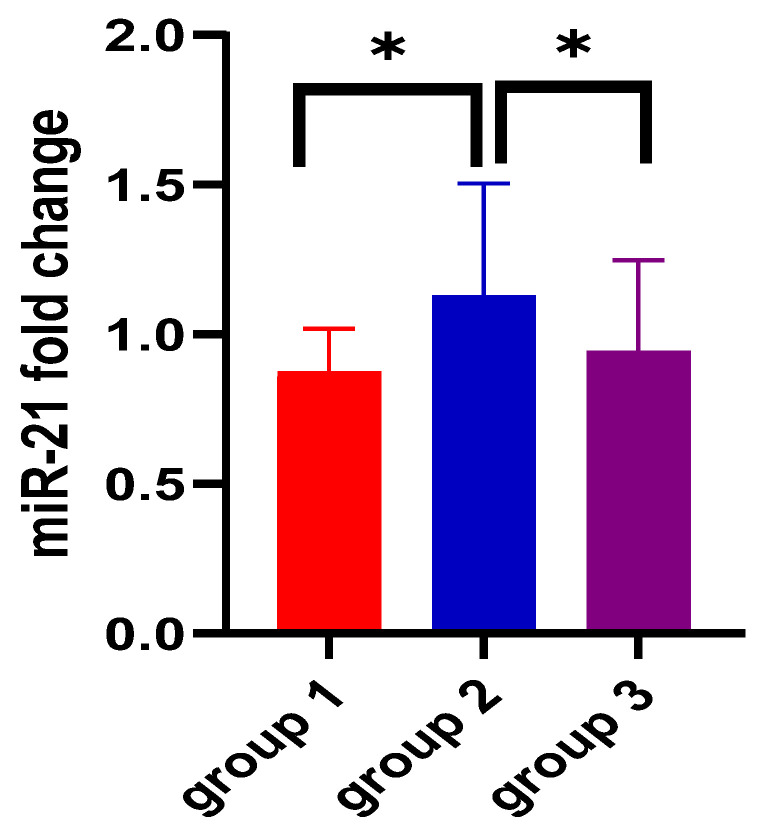
miR-21 2^−ΔΔCt^ comparison (expression fold change) among the three groups. Legend: * *p* < 0.05; miR—miRNA; group 1—UC patients; group 2—CDI patients; group 3 (control group)—IBS-D patients.

When assessing miRNA expression variation among the three groups, no significant differences were found for miR-155 either for ΔCt- or for ΔΔCt-derived parameters (*p* = 0.74 and *p* = 0.73, respectively). Figure 1 and Figure 2 also aid in the better visualization of the similar values found for miR-155, compared between the three groups. However, miR-21 ΔCt levels presented significantly higher values among the ulcerative colitis group (*p* < 0.01), and the most important expression fold change was noticed in patients with C. difficile colitis. Figure 3 and Figure 4 also show the significant miR-21 expression variation between groups 1 and 2, and group 2 and control. Therefore, we further sought to conduct a ROC analysis and assessed how miR-21 can be used to distinguish patients from groups 1 and 2 from patients belonging to the control group, suffering from a functional gastrointestinal disorder. These results are represented in Table 3. Although miR-21 expression could aid in the differential diagnosis between group 1 and group 2 (*p* = 0.02), and group 2 and group 3 (*p* = 0.03), respectively, the obtained cut-off values presented unsatisfactory and imbalanced sensitivity and specificity values.

A correlation between miR-155 and miR-21 expression and the parameters of ulcerative colitis severity (UCDAI, extension degree, and severity assessed based on UCDAI) was further investigated, but no significant results were found. Similarly, non-significant results were obtained when assessing a possible relationship between miR-155/miR-21 expression and fecal calprotectin levels. These findings are further detailed through Table 4.

## 4. Discussion

Despite significant progress in recent years, the etiology of ulcerative colitis (UC) remains poorly understood [1,2]. Likewise, the pathological mechanisms are not fully elucidated. The gold standard for diagnosis remains colonoscopy and histopathological examination [1,2], which is invasive and can present significant adverse effects, and hence the need to identify new biomarkers to facilitate positive diagnosis and improve patient outcomes. Lately, attention has been focused on microRNAs (miRs), especially miR-155 and miR-21 [31]. MiRs are small molecules [32] that have the advantage of being obtained by relatively minimally invasive procedures, are stable in peripheral blood and tissues, and can be rapidly quantified using real-time PCR or microarrays [16,17]. Therefore, the potential utility of circulating miRs as biomarkers has become a promising possibility and a goal of this research. There are currently 2500 mature human miRNA transcripts [33]. Recent studies have shown distinct miR expression profiles for tissue [17], plasma/serum [17,18,27], and feces [31].

miR-155 is one of the most studied miRNAs [17]. Target genes regulated by miR-155 include approximately 140 genes that encode for immunomodulatory proteins, tumor-suppressor proteins, and inflammatory-related proteins [32]. The dysregulation of miR-155 is implicated in Helicobacter pylori-related gastric disease in adults [34] and children [35], as well as in gastroduodenal ulcer [36]. Its expression can be downregulated by targeting Smad2 and inhibiting gastric cancer cell metastasis [37] and upregulated in colorectal cancer, promoting proliferation and invasion, and it is closely related to tumor stage [38]. In this study, we found that the expression levels of miR-155-5p were almost the same for the two conditions and the control group (UC: 4.22 ± 1.61, CDI: 3.94 ± 1.62, IBS-D: 4.26 ± 1.26). When assessing miRNA expression variation between the three groups, no significant differences were found for miR-155 either for ΔCt- or for ΔΔCt-derived parameters (*p* = 0.74 and *p* = 0.73, respectively). This means that miR-155 is expressed almost the same in the two diseases (UC and CDI) and in the patients included in the control group represented by patients with IBS-D. These findings are consistent with previous studies which also reveled possible courses of action and showed that miR-155 is upregulated in UC patients by targeting the suppressor of cytokine signaling 1 (SOCS1), which is an inhibitor of inflammation [26], or by downregulating the expression of FOXO3a [18,25]. According to the research of Paraskevi et al., miR-155 is upregulated in UC 7.82 ± 1.2 fold change compared to healthy controls [18]. For CDI, there are only two references that correlate it with miR-155. The first results from colonic tissue, from an in vivo murine model, which pointed out that miR-155 may have a role in regulating the host’s immune response during early- and late-stage CDI [39], and the second, Alanis et al., showed that Clostridioides difficile initiation factor 1 can be regulated by binding to both microRNA-155 and microRNA-146 [40]. Finally, for irritable bowel syndrome, miR-155 plays an important role in intestinal mucosal barrier function and modulating intestinal inflammation by activating innate immune-related pathways, which leads to visceral hypersensitivity and diarrhea in IBS-D patients [22]. To the best of our knowledge, we did not find any articles in the literature comparing the circulating expression of miR-155 in patients with UC and IBS-D or with CDI.

MiR-21-5p is among the most abundant and highly conserved miRNAs recognized [41]. Functionally, it has been assigned a variety of activities [41]. It is involved in the regulation of several cardiovascular diseases: myocardial infarction [42], multiple sclerosis [42], and complications of type 2 diabetes [43], as well as colorectal cancer [19] and gastric cancer [42]. In our study, miR-21 ΔCt levels presented significantly higher values among the ulcerative colitis group (*p* < 0.01), and the most important expression fold change was noticed in patients with CDI, suggesting that the variation of miR-21 plasma expression was found in all three studied groups but is higher in patients with UC and CDI, compared to the control group represented by patients with IBS-D. These results were similar to previous studies that reported an increased expression of miR-21 in in the peripheral blood of UC patients [18,19,44]. Paraskevi et al. demonstrated that miR-21 is upregulated by 5.29 ± 0.34 fold compered to healthy controls [18]. Unlike our results, Schaefer et al. obtained that circulating miR-21 levels are statistically significantly reduced compared with non-IBD patients [45]. Regarding CDI and IBS-D, we did not find studies that directly present the link with miR-21. However, Nakata et al. described in an animal model, on mice, that commensal bacteria (including Clostridium difficile, Helicobacter pylori, and Campilobacter jejuni) increase the miR-21 expression level and facilitate the permeability of intestinal epithelial cells [37,46]. Finally, miR-21 expression induces ADP ribosylation factor 4, which impairs intestinal barrier functions through the regulation of tight junction proteins such as claudin-4 and occludin [46], and which can be associated with IBS-D pathogenesis [22].

The ROC analysis showed that miR-21 expression could aid in the differential diagnosis between group 1 (UC) and group 2 (CDI) (*p* = 0.02) or group 2 (CDI) and the control group (IBS-D) (*p* = 0.03), respectively, but the cut-off values obtained presented unsatisfactory and imbalanced sensitivity and specificity values. To our knowledge, this is the first study that compares circulating miR-21 levels between UC and CDI, and between CDI and the IBS-D group. Unfortunately we did not obtain a statistically significant difference between group 1 (UC) and the control group (IBS-D) (*p* = 0.99). In contrast to our research, Hassan et al., in a study comprising 100 patients, found that miR-21 was significantly higher in active UC when compared to IBS and was helpful in differentiating UC from non-UC patients (AUC was 0.844, with a sensitivity of 87.5% and a specificity of 91.7%) [19]. On the other hand, in a meta-analysis published by Yan et al., the results obtained were similar to ours, not showing a statistically significant difference between UC patients and non-IBD patients (SMD = 6.67, CI = 3.75–9.58) [47]. Looking at the differentiation of UC and other forms of intestinal inflammation, such as Crohn’s disease, circulating miR-155 seems to be a very good biomarker [18,24,45]; instead, circulating miR-21 was found to be upregulated [18,24] and downregulated [24,45,47] in both diseases. According to this study, miR-155 and miR-21 cannot be considered markers of disease progression, as they do not show a significant association with the extension (*p* = 0.63, and *p* = 0.88) and severity (*p* = 0.97 and 0.34) of the disease. Concerning the severity of colitis, Yan et al., in a meta-analysis involving five studies, demonstrated that tissue miR-21 expression was significantly increased in patients with active UC compared to those in remission (SMD = 2.97, CI = 0.40–5.53) [47]. Similarly to our findings, but with other miRs, Wu et al., in a study with a small number of patients, highlighted that miR-103-2* and miR-362-3p showed the highest increase in expression in active UC patients with 3.1 and 5.2 times, respectively. However, no difference in the expression of miRNAs was detected concerning the extension of the disease (pancolitis and left-sided colitis subgroups) [20]. In another study, involving 30 patients with active UC, which evaluated miR-16, no statistically significant association was found with the severity of the assessed condition (according to Truelove and Witts criteria), nor with the overall assessment of endoscopic severity, *p* = 0.90, assessed using the total score of Ulcerative Colitis Endoscopic Index of Severity [48].

Further studies are needed to elucidate the role of a miRs in certain tissues, cell types [49], peripheral blood, or other body fluids, because different biological products may have different expressions for the same miRs [31]. In feces, miR expression may be a much more accurate tool for assessing disease activity or mucosal healing in gastrointestinal diseases due to localized changes and the lack of involvement of other structures (e.g., erythrocytes or leukocytes, which could express the same miRs) [31]. Similarly, miR-155, which is expressed in lymphocytes, is involved in the regulation of mucosal inflammation [32,34]. miR-21 expression in peripheral blood is less represented than the determination made from colon tissue [47]. In this direction, in a study on a very small number of patients, Takagi et al. showed that miR-155 and miR-21 collected from the colonic mucosa were highly expressed in patients with active ulcerative colitis, compared to healthy volunteers [44]. Similarly, Wu et al. found that, in the tissue of UC patients, miR-21 was upregulated by 354.6% compared to controls [20]. Several studies have shown that miR-21 is even more expressed with the progression from normal mucosa to IBD to dysplasia/neoplasia associated with IBD [15,42,50]. Therefore, the increase in the expression of this miR could indicate a malignant transformation [19]. Currently, the monitoring of dysplasia occurrence in patients with UC is performed based on the associated risk using chromoendoscopy with or without magnification and targeted biopsies [2]. Therefore, highlighting this progression by measuring miR-21 expression would yield significant benefits.

Although the results for miR-21 are promising, given the fact that miR-21 and -155 are involved in the pathophysiology of dozens of diseases, they cannot be specific biomarkers for any disease [41]. Finding a solution whereby miRNAs biomarkers in UC are not interfered with by other diseases or therapies is a future challenge [51]. Due to the known effects of miR-155 and miR-21 on the modulation of intestinal immunity and inflammation, particularly in the pathogenesis of UC, IBS-D, and ICD, they can be candidates to modulate the inflammatory/malignant cascade or for future therapeutic usage [32,41], similar to Miravirsen, which is an antimir used for treating the hepatitis C virus (HCV) by inhibiting miR-122-HCV [52].

As with the majority of studies, the design of the current study is subject to limitations. The first limitation of this study was its small sample size (especially for the CDI cohort) and matching for age and gender was not possible. Secondly, we did not include a control group of healthy individuals because it was difficult to perform colonoscopies on healthy people to exclude diseases without clinical noise such as colitis of any etiology, polyps, and small tumors. Thirdly, the possibility of an overlap (circulating miR-155 and -21) is high due to the presence of intestinal inflammation or another clinically silent concomitant disease. Finally, we need a unified measurement platform and the normalization of the acquired data.

## 5. Conclusions

Circulating miR-155 is upregulated in UC, CDI, and IBS-D, but it cannot differentiate among them. As for circulating miR-21, it is significantly more upregulated in patients with CDI and UC, but the cut-off values are unsatisfactory, and the sensitivity and specificity values are imbalanced. Based on the results obtained in this study, miR-155-5p and miR-21 cannot be used as specific biomarkers for UC and they cannot differentiate between UC, IBS-D, and CDI. However, they can be considered as potential preventive and therapeutic targets that could enhance the intestinal epithelial barrier and/or modulate mucosal immune response, facilitating the development of personalized treatment strategies and possibly the early detection of malignant transformation.

Detecting the presence of miR-155 and miR-21 in patients with Clostridioides difficile infection and irritable bowel syndrome opens up new research possibilities for modulating the inflammatory cascade and/or therapeutic intervention, and these microRNAs can be developed as pharmacodynamic biomarkers for use in clinical care. Further studies are needed to validate these results.

## Figures and Tables

**Table 1:** Demographic characteristics of the population included in the study.

Parameter	Group 1 (*n* = 34) (Mean ± SD)	Group 2 (*n* = 17) (Mean ± SD)	Control Group (*n* = 33) (Mean ± SD)	*p* Value
Age (years)	45.91 ± 15.23	65 ± 12.51	59.18 ± 13.86	<0.0001
Gender (*n*) Female Male	12 22	8 9	25 8	<0.0001
Background (*n*) Urban Rural	24 10	8 9	15 18	0.08

Legend: *n*—number; SD—standard deviation.

**Table 2:** Paraclinical data and miRNA expression patterns of the population included in the study.

Parameter	Group 1 (*n* = 34) (Mean ± SD)	Group 2 (*n* = 17) (Mean ± SD)	Control Group (*n* = 33) (Mean ± SD)	*p* Value
Hemoglobin (g/dL)	11.32 ± 1.87	11.82 ± 1.25	12.52 ± 0.92	<0.0001
Leukocytes * (cells/µL)	10205 ± 2275	12182 ± 2776	8473 ± 1847	<0.0001
Serum iron (µg/dL)	54.24 ± 24.02	52.94 ± 13.44	63.09 ± 15.14	0.14
Albumin (g/L)	34.47 ± 2.60	36.24 ± 3.54	37.21 ± 1.83	<0.01
Fecal calprotectin (µg/mg)	551.5 ± 424.1	339.4 ± 125.7	49.82 ± 22.22	<0.0001
C reactive protein (mg/dL)	2.29 ± 1.42	5.18 ± 1.82	0.36 ± 0.16	<0.0001
miR-155 ΔCt	4.22 ± 1.61	3.94 ± 1.62	4.26 ± 1.26	0.74
miR-155 2^−ΔΔCt^	4.11 ± 8.46	4.94 ± 9.68	2.83 ± 5.41	0.73
miR-21 ΔCt	0.05 ± 0.34	−0.27 ± 0.41	−0.04 ± 0.27	<0.0001
miR-21 2^−ΔΔCt^	0.87 ± 0.14	1.12 ± 0.37	0.94 ± 0.30	<0.0001

Legend: Ct—cycle threshold; *n*—number; SD—standard deviation. * Brown–Forsythe and Welch ANOVA tests were applied.

**Table 3:** ROC analysis of miR-21 expression for distinction among the study subgroups.

Parameter	Compared Groups	AUC (95% CI)	Cut-off	Sensitivity (95% CI)	Specificity (95% CI)	*p* Value
miR-21 ΔCt	group 1 vs. control group	0.50 (0.36–0.64)	−0.99	100% (89.85–100%)	3.03% (0.15–15.32%)	0.99
	group 2 vs. control group	0.68 (0.51–0.85)	−0.29	35.29% (17.31–58.70%)	96.97% (84.68–99.84%)	0.03
	group 1 vs. group 2	0.69 (0.52–0.85)	−0.06	88.24% (73.38–95.33%)	41.18% (21.61–63.99%)	0.02
miR-21 2^−ΔΔCt^	group 1 vs. control group	0.50 (0.36–0.64)	0.82	100% (89.57–100%)	8.82% (3.04–22.96%)	0.99
	group 2 vs. control group	0.68 (0.51–0.85)	0.92	96.97% (84.68–99.84%)	41.18% (21.61–63.99%)	0.03
	group 1 vs. group 2	0.69 (0.52–0.85)	0.92	88.24% (73.38–95.33%)	41.18% (21.61–63.99%)	0.02

**Table 4:** Non-parametric Spearman correlation for assessment of the relationship between the studied miRNA expressions and parameters of ulcerative colitis severity/fecal calprotectin levels.

Independent Variable	Dependent Variable	Spearman’s Rank Correlation Coefficient	*p* Value
miR-155 ΔCt	UCDAI	−0.005	0.97
	UC extension	−0.084	0.63
	UC severity	−0.716	0.68
	fecal calprotectin	0.070	0.52
miR-21 ΔCt	UCDAI	−0.168	0.34
	UC extension	0.026	0.88
	UC severity	−0.169	0.33
	fecal calprotectin	0.060	0.73

Legend: Ct: cycle threshold; miR—miRNA; UC—ulcerative colitis; UCDAI—ulcerative colitis disease activity index.

## Data Availability

Data are contained within the article.

## References

[1] Mihai C., Cijevschi-Prelipcean C., Diculescu M. (2023). Rectocolita ulcero-hemoragică (Colita ulcerativă). Gastroenterologie si Hepatologie Clinica.

[2] Maaser C., Sturm A., Vavricka S.R., Kucharzik T., Fiorino G., Annese V., Calabrese E., Baumgart D.C., Bettenworth D., Borralho Nunes P. (2019). European Crohn’s and Colitis Organisation [ECCO] and the European Society of Gastrointestinal and Abdominal Radiology [ESGAR]. ECCO-ESGAR Guideline for Diagnostic Assessment in IBD Part 1: Initial diagnosis, monitoring of known IBD, detection of complications. J. Crohn’s Colitis.

[3] Kelly C.R., Fischer M., Allegretti J.R., LaPlante K., Stewart D.B., Limketkai B.N., Stollman N.H. (2021). ACG Clinical Guidelines: Prevention, Diagnosis, and Treatment of Clostridioides difficile Infections. Am. J. Gastroenterol..

[4] Joshi N.M., Marks I.H., Crowson R., Ball D., Rampton D.S. (2017). Incidence and outcome of clostridium difficile infection in hospitalized patients with inflammatory bowel disease in the UK. J. Crohn’s Colitis.

[5] Clayton E.M., Rea M.C., Shanahan F., Quigley E.M.M., Kiely B., Hill C., Ross R.P. (2009). The vexed relationship between clostridium difficile and inflammatory bowel disease: An assessment of carriage in an outpatient setting among patients in remission. Am. J. Gastroenterol..

[6] Bishop E.J., Tiruvoipati R. (2022). Management of Clostridioides difficile infection in adults and challenges in clinical practice: Review and comparison of current IDSA/SHEA, ESCMID and ASID guidelines. J. Antimicrob. Chemother..

[7] INSP. https://insp.gov.ro/download/ghid-diagnostic-tratament-si-prevenire-clostridium-difficile-pdf/.

[8] Szałwińska P., Włodarczyk J., Spinelli A., Fichna J., Włodarczyk M. (2021). IBS-Symptoms in IBD Patients—Manifestation of Concomitant or Different Entities. J. Clin. Med..

[9] Quigley E.M. (2016). Overlapping irritable bowel syndrome and inflammatory bowel disease: Less to this than meets the eye?. Therap. Adv. Gastroenterol..

[10] Drossman D.A., Hasler W.L. (2016). Rome IV-Functional GI Disorders: Disorders of Gut-Brain Interaction. Gastroenterology.

[11] Lacy B.E., Patel N.K. (2017). Rome Criteria and a Diagnostic Approach to Irritable Bowel Syndrome. J. Clin. Med..

[12] Cioffi M., Rosa A.D., Serao R., Picone I., Vietri M.T. (2015). Laboratory markers in ulcerative colitis: Current insights and future advances. World J. Gastrointest. Pathophysiol..

[13] Wen B.J., Te L.G., Liu X.X., Zhao J.H. (2022). The value of fecal calprotectin in Clostridioides difficile infection: A systematic review. Front. Physiol..

[14] Onişor D., Boeriu A., Pascarenco O., Brusnic O., Dobru D. (2018). Role of fecal calprotectin as a biomarker of intestinal inflammation in ulcerative colitis: A prospective study. Rev. Romana Med. Lab..

[15] James J.P., Riis L.B., Malham M., Høgdall E., Langholz E., Nielsen B.S. (2020). MicroRNA Biomarkers in IBD—Differential Diagnosis and Prediction of Colitis-Associated Cancer. Int. J. Mol. Sci..

[16] Boicean A., Birsan S., Ichim C., Boeras I., Roman-Filip I., Blanca G., Bacila C., Fleaca R.S., Dura H., Roman-Filip C. (2023). Has-miR-129-5p’s Involvement in Different Disorders, from Digestive Cancer to Neurodegenerative Diseases. Biomedicines.

[17] Chapman C.G., Pekow J. (2015). The emerging role of miRNAs in inflammatory bowel disease: A review. Therap. Adv. Gastroenterol..

[18] Paraskevi A., Theodoropoulos G., Papaconstantinou I., Mantzaris G., Nikiteas N., Gazouli M. (2012). Circulating MicroRNA in inflammatory bowel disease. J. Crohn’s Colitis.

[19] Ahmed Hassan E., El-Din Abd El-Rehim A.S., Mohammed Kholef E.F., Abd-Elgwad Elsewify W. (2020). Potential role of plasma miR-21 and miR-92a in distinguishing between irritable bowel syndrome, ulcerative colitis, and colorectal cancer. Gastroenterol. Hepatol. Bed. Bench..

[20] Wu F., Guo N.J., Tian H., Marohn M., Gearhart S., Bayless T.M., Brant S.R., Kwon J.H. (2011). Peripheral blood microRNAs distinguish active ulcerative colitis and Crohn’s disease. Inflamm. Bowel Dis..

[21] Fasseu M., Tréton X., Guichard C., Pedruzzi E., Cazals-Hatem D., Richard C., Aparicio T., Daniel F., Soulé J.C., Moreau R. (2010). Identification of restricted subsets of mature microRNA abnormally expressed in inactive colonic mucosa of patients with inflammatory bowel disease. PLoS ONE.

[22] Guo J.G., Rao Y.F., Jiang J., Li X., Zhu S.M. (2023). MicroRNA-155-5p inhibition alleviates irritable bowel syndrome by increasing claudin-1 and ZO-1 expression. Ann. Transl. Med..

[23] Martínez C., Rodiño-Janeiro B.K., Lobo B., Stanifer M.L., Klaus B., Granzow M., González-Castro A.M., Salvo-Romero E., Alonso-Cotoner C., Pigrau M. (2017). miR-16 and miR-125b are involved in barrier function dysregulation through the modulation of claudin-2 and cingulin expression in the jejunum in IBS with diarrhoea. Gut.

[24] Síbia C.d.F.d., Quaglio A.E.V., Oliveira E.C.S.d., Pereira J.N., Ariede J.R., Lapa R.M.L., Severino F.E., Reis P.P., Sassaki L.Y., Saad-Hossne R. (2024). microRNA–mRNA Networks Linked to Inflammation and Immune System Regulation in Inflammatory Bowel Disease. Biomedicines.

[25] Min M., Peng L., Yang Y., Guo M., Wang W., Sun G. (2014). MicroRNA-155 is involved in the pathogenesis of ulcerative colitis by targeting FOXO3a. Inflamm. Bowel Dis..

[26] Pathak S., Grillo A.R., Scarpa M., Brun P., D’Incà R., Nai L., Banerjee A., Cavallo D., Barzon L., Palù G. (2015). MiR-155 modulates the inflammatory phenotype of intestinal myofibroblasts by targeting SOCS1 in ulcerative colitis. Exp. Mol. Med..

[27] Yang Y., Ma Y., Shi C., Chen H., Zhang H., Chen N., Zhang P., Wang F., Yang J., Yang J. (2013). Overexpression of miR-21 in patients with ulcerative colitis impairs intestinal epithelial barrier function through targeting the Rho GTPase RhoB. Biochem. Biophys. Res. Commun..

[28] Satsangi J., Silverberg M.S., Vermeire S., Colombel J.F. (2006). The Montreal classification of inflammatory bowel disease: Controversies, consensus, and implications. Gut.

[29] Song J., Bai Z., Han W., Zhang J., Meng H., Bi J., Ma X., Han S., Zhang Z. (2012). Identification of suitable reference genes for qPCR analysis of serum microRNA in gastric cancer patients. Dig. Dis. Sci..

[30] Livak K.J., Schmittgen T.D. (2001). Analysis of relative gene expression data using real-time quantitative PCR and the 2(-Delta Delta C(T)) Method. Methods.

[31] Schönauen K., Le N., von Arnim U., Schulz C., Malfertheiner P., Link A. (2018). Circulating and Fecal microRNAs as Biomarkers for Inflammatory Bowel Diseases. Inflamm. Bowel Dis..

[32] Wan J., Xia L., Xu W., Lu N. (2016). Expression and Function of miR-155 in Diseases of the Gastrointestinal Tract. Int. J. Mol. Sci..

[33] miRBase. https://www.mirbase.org.

[34] Oertli M., Engler D.B., Kohler E., Koch M., Meyer T.F., Muller A. (2011). MicroRNA-155 is essential for the T cell-mediated control of Helicobacter pyloriinfection and for the induction of chronic Gastritis and Colitis. J. Immunol..

[35] Oana S.M., Claudia B., Lelia R.A., Simona M., Claudia C., Daniela D.E. (2022). Differential Expression of Tissular miRNA-155 in Pediatric Gastritis. J. Clin. Med..

[36] Cheng S.F., Li L., Wang L.M. (2015). miR-155 and miR-146b negatively regulates IL6 in Helicobacter pylori (cagA+) infected gastroduodenal ulcer. Eur. Rev. Med. Pharmacol. Sci..

[37] Li R., Hu Y., Hou S. (2022). An Exploration of Oral-Gut Pathogens Mediating Immune Escape of Pancreatic Cancer via miR-21/PTEN Axis. Front. Microbiol..

[38] Qu Y.L., Wang H.F., Sun Z.Q., Tang Y., Han X.N., Yu X.B., Liu K. (2015). Up-regulated miR-155–5p promotes cell proliferation, invasion and metastasis in colorectal carcinoma. Int. J. Clin. Exp. Pathol..

[39] Kennedy K.F. (2016). The Effects of Surface Layer Proteins Isolated from Clostridium Difficile on TLR4 Signalling. Ph.D. Thesis.

[40] Alanis E.L. (2022). Targeting Protein Synthesis in Clostriodioides Difficile to Develop Antimicrobial Candidate. Master’s Thesis.

[41] Jenike A.E., Halushka M.K. (2021). miR-21: A non-specific biomarker of all maladies. Biomark. Res..

[42] Nguyen H.T., Kacimi S.E.O., Nguyen T.L., Suman K.H., Lemus-Martin R., Saleem H., Do D.N. (2021). MiR-21 in the Cancers of the Digestive System and Its Potential Role as a Diagnostic, Predictive, and Therapeutic Biomarker. Biology.

[43] Olivieri F., Spazzafumo Bonafè M., Recchioni R., Prattichizzo F., Marcheselli F., Micolucci L., Mensà E., Giuliani A., Santini G., Gobbi M. (2015). MiR-21-5p and miR-126a-3p levels in plasma and circulating angiogenic cells: Relationship with type 2 diabetes complications. Oncotarget.

[44] Takagi T., Naito Y., Mizushima K., Hirata I., Yagi N., Tomatsuri N., Yoshikawa T. (2010). Increased expression of microRNA in the inflamed colonic mucosa of patients with active ulcerative colitis. J. Gastroenterol. Hepatol..

[45] Schaefer J.S., Attumi T., Opekun A.R., Abraham B., Hou J., Shelby H., Graham D.Y., Streckfus C., Klein J.R. (2015). MicroRNA signatures differentiate Crohn’s disease from ulcerative colitis. BMC Immunol..

[46] Nakata K., Sugi Y., Narabayashi H., Kobayakawa T., Nakanishi Y., Tsuda M., Hosono A., Kaminogawa S., Hanazawa S., Takahashi K. (2017). Commensal microbiota-induced microRNA modulates intestinal epithelial permeability through the small GTPase ARF4. J. Biol. Chem..

[47] Yan H., Zhang X., Xu Y. (2020). Aberrant expression of miR-21 in patients with inflammatory bowel disease: A protocol for systematic review and meta analysis. Medicine.

[48] Hussein M.S., Ezzat A., Nahed B., Mohamed E.l., Eman T. (2020). Serum expression of microRNA-16 in a cohort of Egyptian patients with ulcerative colitis and its correlation with disease extent and severity. J. Coloproctol..

[49] Whiteoak S.R., Felwick R., Sanchez-Elsner T., Fraser Cummings J.R. (2015). MicroRNAs in Inflammatory Bowel Diseases: Paradoxes and Possibilities. Inflamm. Bowel Dis..

[50] Ludwig K., Fassan M., Mescoli C., Pizzi M., Balistreri M., Albertoni L., Pucciarelli S., Scarpa M., Sturniolo G.C., Angriman I. (2013). PDCD4/miR-21 dysregulation in inflammatory bowel disease-associated carcinogenesis. Virchows Arch..

[51] Kalla R., Adams A.T., Ventham N.T., A Kennedy N., White R., Clarke C., Ivens A., Bergemalm D., Vatn S., Lopez-Jimena B. (2020). Whole Blood Profiling of T-Cell Derived miRNA Allows the Development of Prognostic Models in Inflammatory Bowel Disease. J. Crohn’s Colitis.

[52] Krishnachaitanya S.S., Liu M., Fujise K., Li Q. (2022). MicroRNAs in Inflammatory Bowel Disease and Its Complications. Int. J. Mol. Sci..

